# ‘More extraordinary than mundane … ’ A phenomenological analysis of the experiences of individuals living with CLE and their taking care in the sun

**DOI:** 10.1177/0961203320958067

**Published:** 2020-09-17

**Authors:** Bláithín McGarry, Donal O'Kane, Collette McCourt, Gerard J Gormley

**Affiliations:** 1 Centre for Medical Education, Queen’s University Belfast, Belfast UK; 2 Department of Dermatology, Belfast Health and Social Care Trust, Belfast, UK

**Keywords:** Cutaneous lupus erythematosus, photoprotection

## Abstract

**Introduction:**

CLE is a chronic inflammatory autoimmune condition of which photosensitivity is a major symptom. Individuals living with CLE are advised to practice photoprotection. Despite the benefits for disease control, many individuals living with CLE do not practice optimal photoprotection. The aim of this study was to gain a deep insight into the lived experiences of individuals with CLE and their photoprotective practices.

**Methods:**

A qualitative study approach was conducted, using Hermeneutic phenomenology. Individuals living with CLE were recruited and interviewed. Rich pictures were used to enrich the interviews. Interviews were transcribed and analysed using Template Analysis.

**Results:**

Analysis revealed four themes: ‘Much more than just a photosensitive skin condition’, ‘The impact of sun on CLE and social dynamics’, ‘Drifting to the sun: personal transitions and social norms’ and ‘Taking care in the sun: easier said than done’.

**Discussion and conclusion:**

This study provides a nuanced insight into the lived experiences of individuals with CLE and their photoprotective practices. Taking care in the sun is not a simplistic process. Beyond the biomedical model of illness, the social impact that CLE has on individuals has a dominant influence on their photoprotective behaviours. Such insights could help healthcare professionals tailor photoprotective advice.

## Introduction

Photosensitivity relates to a wide range of conditions caused or exacerbated by sources of ultraviolet radiation (UVR) including sun exposure, sunbeds and certain artificial light sources.^1^ Cutaneous lupus erythematosus (CLE) is a photosensitive autoimmune connective tissue disease with an incidence of 4 per 100,000 of the population.^2^ Affecting more women than men, CLE can have a profound impact on quality of life.^3,4^ For CLE patients, exposure to UVR may exacerbate skin and systemic symptoms if they have co-existing systemic lupus erythematosus (SLE).^5^ In addition, UVR can have a negative psychological impact on wellbeing including anxiety and depressive related symptoms.^6^

The propensity of photosensitivity related CLE flares can be reduced by effective photoprotection.^5^ Photoprotective measures include applying sunscreen, seeking shade, avoiding sun between 10am-4pm, and wearing items such as long-sleeved clothing and a wide brimmed hat.^5,7^ A broad-spectrum sunscreen with a minimum sun protection factor (SPF) of 30 should be used daily,^5^ adhering to the ‘tea-spoon’ rule.^8^ Several studies have demonstrated the efficacy of sunscreen in reducing CLE symptoms.^9–12^

Despite the possible benefits for disease control, many CLE patients do not practice adequate photoprotection.^7,13^ Potential reasons for this include forgetfulness, inconvenience and ineffectiveness in preventing flare-ups as cited by one recent study.^14^

Gaining greater insights as to why individuals with CLE do not adhere to photoprotective measures may help guide optimisation of photoprotective behaviours. This has the potential to reduce UVR-related cutaneous and systemic flare-ups. Qualitative research is highly suited to shed light on such factors and help target areas for future research and interventions. Therefore, the aim of this study was to gain a deep understanding of the lived experience of individuals with CLE taking care in the sun.

## Methods

### Ethical approval and quality assurance

Ethical approval was granted by East Midlands-Leicester South Research Ethics Committee (REC reference: 20/EM/0019). Written informed consent was obtained from all participants. The project adhered to the *Consolidated criteria for Reporting Qualitative research* checklist.^15^

### Conceptual orientation of study

The aim of our research was to gain a deep understanding of the lived experiences of CLE patient’s photoprotective behaviours. Not only to explore the more *explicit* experiences but also the more *implicit* experiences. A number of qualitative research methodological approaches can facilitate gaining such insights e.g. narrative based research. However, in our study we were keen to not only gain insights to patient’s experiences but also the influences that shaped these experiences. On this basis, we chose a hermeneutic phenomenology approach in our study.^16^ Phenomenology aims to capture how a phenomena (in this case CLE patients' photoprotective behaviours) is experienced in the consciousness of such individuals. Moreover, in hermeneutic phenomenology, this approach takes cognisant of the contextual influences that shape such patient experiences.

### Setting, sampling and recruitment

Patients were recruited from the Department of Dermatology, Belfast Health and Social Care Trust, Northern Ireland. Phenomenological studies typically aim to strike a balance between the deep understanding of participants’ experiences and the broader insights gained by sampling a larger number of participants (and not being oversaturated by the volume of data).^17^ Therefore, the research team aimed to recruit up to 10 participants depending on data saturation. Adult patients with a formal diagnosis of CLE were identified by two consultant dermatologists and invited to participate in the study. A convenience sampling strategy was used.

### Data collection

Exploratory one-on-one qualitative interviews, either face-to-face or via telephone, were carried out by BMcG who received training in such interview techniques. In accordance with phenomenology, interviews were minimally structured to allow emerging themes to remain as true as possible to participants experiences.

Verbal interview data was enhanced by rich pictures, a visual tool which can provide insight into a participant’s experience of a phenomena.^18^ The use of rich pictures acknowledges that some individuals may be less verbally astute and unable to fully express their experiences by words alone. This enables researchers to gain deeper insights into complex phenomena such as human behaviours.

**Figure 1.**
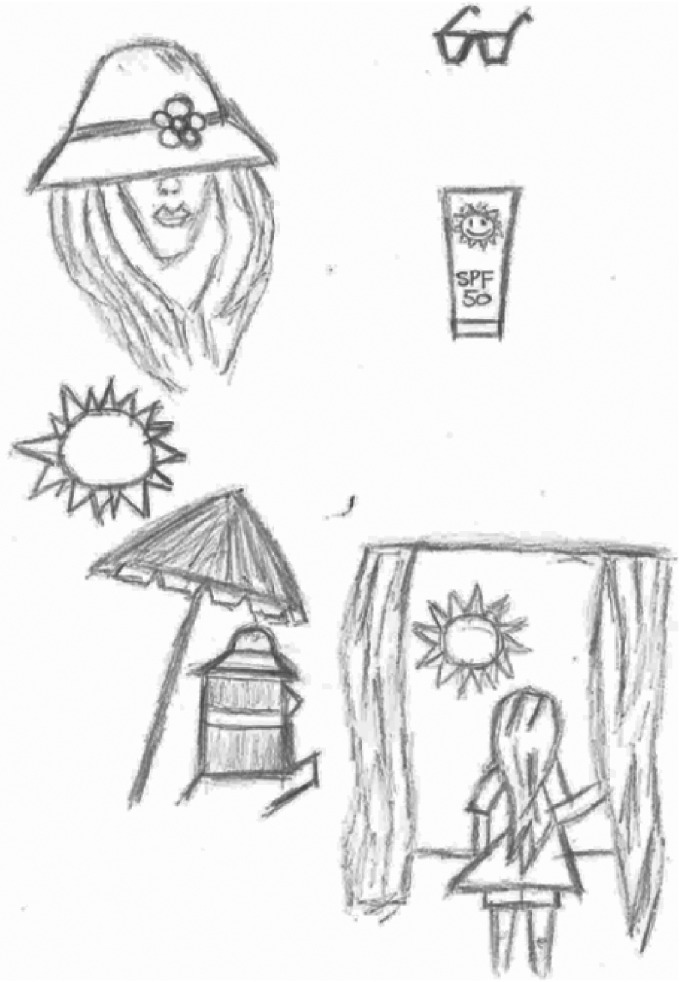
Example of a rich picture drawn by a research participant (Kate*) *pseudonym.

To begin the interview, participants were invited to draw a rich picture representing their experience of living with CLE, the impact the sun has on their condition and their experiences of taking care in the sun (See Figure 1 for an example). Participants who conducted a telephone interview completed this picture, with guidance, and emailed the image to the interviewer prior to the interview.

Pre-planned interview questions followed, exploring participant’s experiences of photoprotection. Further unplanned questions were asked to explore their experiences in greater depth. Data collection ceased when the researchers believed they had data sufficiency. All interviews were transcribed verbatim, checked for accuracy and anonymised using pseudonyms.

### Data analysis

Interview transcripts provided the focus for analysis. The rich pictures were not analysed directly but used to support analysis. A template analysis approach was used to analyse the data because of its fit with hermeneutic phenomenology.^19^ This permitted a structured approach of participants’ reported experiences, researchers’ interpretations and how the data contributed to the whole understanding of the experience of practicing photoprotection as a CLE patient (i.e. the ‘hermeneutic circle’).^19^

The entire research team contributed to the analysis. To begin analysis, tentative *a priori* codes were identified based on the research aim. *A priori* codes were applied to identify relevant text in one transcript. *A priori* codes were redefined or excluded, or new *a priori* codes were added after reading the transcript. *A priori* codes were then organised into clusters to identify *preliminary themes* and used to develop an initial template. This initial template was applied to the remaining transcripts and progressively modified. To conclude analysis, all transcripts were coded against the final template. Member checking of the results was conducted to seek respondent validation. The research team, which consisted of consultant dermatologists, an expert phenomenological researcher and a medical student were continually reflexive (i.e. being continually mindful of potential researcher biases and mitigating for this through critical dialogue and writing) throughout the study. Analysis ended when all of the researchers agreed that a thick and rich description had been achieved.

## Results

Ten participants took part in the study and generated 376 minutes of interview data. Analysis yielded four themes: *1) ‘Much more than just a photosensitive skin condition’, 2) ‘The impact of sun on CLE and social dynamics’, 3) ‘Drifting to the sun’: personal transitions and social norms* and *4) Taking care in the sun: easier said than done?*

### ‘Much more than *just* a photosensitive skin condition’

Participant’s illness experiences of CLE were central to their photoprotection adherence. Often participants recounted that CLE was perceived as ‘just a skin condition’ by others (including health professionals) resulting in false assumptions about their condition. Whilst exposure to the sun could exacerbate their skin symptoms, it could also exacerbate systemic symptoms if they have co-existing SLE.

*“ … when the sun’s triggered me, my muscles would be really painful, my headaches increase, the fatigue increases more”* ***(Kate)***

In addition to such biomedical dimension of illness, participants often experienced psychological and emotional sequalae. It was not uncommon for participants to experience mental health and wellbeing issues.

*“It also contributes to my depression … I take anti-depressants and have done from when I was diagnosed … I think it goes hand in hand because the things that you have to go through, like avoiding the sun constantly”* ***(Sarah)***

### The impact of sun on CLE and social dynamics

#### Skin: An organ of social expression?

Participants experienced significant impact on their social world and interactions as a result of their CLE. Given the photosensitive nature of CLE, involvement of skin sites visible to others, such as one’s face, forearms and hands were typical, with a resultant impact on social interactions. When participants experienced a disease flare in such exposed areas, this could draw unwanted attention.

*“I go into the shop and somebody will come up to me ‘Love, you look awful sick …  are you ok?’  …  because I have a roaring red face with spots … ”* ***(Sarah)***

Conversely, if participants were experiencing an improvement in such exposed areas, this could also draw attention. This could either be a pleasurable experience … 

*“They’ll just say ‘You’re looking good’, ‘It’s great that you haven’t got your rash’ …  that makes me feel good about myself”* ***(Ann)***
…

 or reinforce their fear of experiencing an exacerbation of their CLE.

*“ … everybody would say you’ve got lovely skin for your age, and then that makes me even more conscious if I do get something, you know?”* ***(Kate)***

#### ‘Blemished and social isolating’: The stigma of living with CLE

Having a disease flare on exposed areas promoted a range of participant’s experiences when in the presence of others. Often participants felt *different.* Dependent on the social circumstances, such experiences could have variable impacts. Others who were aware of their CLE would often offer support.

*“Our manager … he has a lot of empathy. He would encourage me ‘Come in if you don’t want to go out the shop front today and go out to the back’”* ***(Ann)***

However, when in the presence of strangers, a disease flare could draw unwelcomed attention with participants often wanting to conceal their condition.

*“The first thing I do when I go in is put my housecoat on, put my hood up because I’m safer when I’m hiding. Even though I’m indoors, my subconscious is trained now to hide.”* ***(Sarah)***

Participants often experienced a desire to exclude themselves from social situations that had potential of sun exposure.

*“ … if there’s a barbeque on everybody’s just going to be out in the back garden, and you think what’s the point in really going because I’m going to be sitting indoors if there’s no shade …  it can make you feel very isolated”* ***(Kate)***

### ‘Drifting to the sun’: *Personal transitions and social norms*

Participants recalled their ‘relationship’ with the sun prior to developing CLE. During this time, sun exposure was a pleasurable and social phenomenon.

*“Just sitting out in the garden … just feeling free in the nice sun …  the air around you … having a wee glass of wine. But now I can’t do that I have to be covered up you know?”* ***(Ann)***

Moreover, participants experienced that enjoying sun exposure was a societal norm despite public health advice regarding photoprotection. Receiving a diagnosis of CLE marked an important transition in how participants experienced the sun. Participants were at various stages of acceptance of their new relationship with the sun. Some were accepting while others had a sense of loss compared to their ‘pre-CLE’ self, experiencing a longing for a time in their life when they could ‘enjoy’ the sun again.

*“I used to get very depressed when I saw people in summer clothes you know shorts, wee tops … even if I have a top on I’ve always to keep covered, I always have to have a shirt on me right down to my wrists.”* ***(Susan)***

Such difficulties in reconciling their need to avoid the sun ran counter-current with their perceived notion of enjoying the sun at a societal level. Participants acknowledged that the prevailing social norm of favouring sun exposure could mediate their desire to test the limits of their condition in the sun.

### Taking care in the sun: *Easier said than done?*

Participants experienced mediating factors that either promoted or inhibited their compliance with photoprotective advice. Such factors were either intrinsic or extrinsic to them as a person. Many of the experiences were nuanced and perhaps considered mundane by others, but important to them.

#### Facilitatory factors experienced in taking care in the sun

A dominant experience for participants was the impact that the sun had on their symptoms. When in anticipation of sun exposure, participants would commonly recall negative experiences of sun exposure in relation to their condition. Reliving such experiences could drive engagement with photoprotective measures. For example, if participants experienced more severe flares in the sun, this promoted a greater desire to adhere to photoprotective advice.

*“That one day I’ve went out with no suncream on, the anxiety in my stomach because I know I’m going to end up sick, because the sun makes me sick”* ***(Sarah)***

Intrinsic drivers could be reinforced by external drivers to take care in the sun, such as photoprotective advice from healthcare professionals. Sustained advice by healthcare professionals had the potential to promote proactivity in participant’s photoprotective practices.

*“They [doctors] always make sure that they give strict instructions when I’m leaving to make sure I’m putting my sunblock on”* ***(Henry)***

In addition to general advice about avoiding sun exposure, the provision of practical assistance promoted a greater desire to adhere to such advice.

*“I would get my suncream on prescription …  which is really good because it’s pretty expensive especially the one I do get”* ***(Kate)***

Recognition of the importance of photoprotection by others who interacted with participants in daily life could promote sustainment of photoprotective measures. For example, support from family and friends who were ‘wise’ to their condition.

*“When we go on holiday they [family] would say ‘Right you sit over there in the shade’ you know? Even my grandchildren god love them they would say to me ‘Nanny now that sun’s too strong, now you have to sit here’* ***(Susan)***

#### Inhibitory factors experienced in taking care in the sun

Participants also experienced a range of factors that suppressed their photoprotective behaviours. Many of these factors could be intrinsic to the individual. The burden of systemic symptoms alongside the extra effort required to adhere to photoprotective advice could inhibit participants photoprotection.

*“It can be a pain, because when you’re not feeling well and you’re tired and fatigued, the thought is just ‘Ugh I have to put this on’”* ***(Kate)***

Furthermore, despite the extra effort taken to adhere to photoprotective measures, participants could still experience the ill effects of sun exposure on their condition, reducing the motivation to continue.

*“ … If I’m in the sun I can just sort of feel it, just sort of tingling like, actually burning you even though you’ve got factor 50 on … why do I bother?”* ***(Kate)***

The continual practical efforts required for adequate photoprotection was another barrier. Participants shared the difficulty of having to continually anticipate the potential of being exposed to the sun and taking preparatory measures in such situations.

*“I feel as if I’m always late for everything, I’m always the last person there because I have to do that much preparing …  after I get washed and that in the morning then I’ve to put my sunblock on, I’ve to wait until that absorbs in then … I put my moisturiser on  … and then my makeup. I feel as if it’s an ordeal some days, I haven’t even the energy to do that you know.”* ***(Susan)***

Furthermore, participants also expressed the practical challenges in adhering to advice relating to sunscreen, long-sleeved clothing and photoprotective clothing. Aside from the time, effort, and for some expense, the unpleasant feeling of engaging with these photoprotective measures was a prominent inhibitory experience.

*“The doctor gave me some stuff but this is like wallpaper paste so I didn’t use that”* ***(Ann)***

Beyond the practical challenges, life and social circumstances could also act as barriers to practicing such photoprotective measures. The demands of daily life, especially if spontaneous, could often override the extra effort it took to instigate photoprotective measures.

*“Now I’ve tried to wear my brimmed hat but like you’re trying to mow a lawn with this hat down over your eyes!”* ***(Ann)***

In certain social circumstances, participants felt a drive to disengage with photoprotective measures in an attempt to not appear different.

*“I would just feel stupid walking along in the summer with an umbrella up. Just drawing attention to yourself even though it I know it would help me”* ***(Kate)***

Finally, whilst healthcare professional advice could promote photoprotective behaviours, often their expectations of how participants should take care in the sun could be unrealistic.

*“One of the things he wanted me to do, I never did it, was to use white cotton gloves when I was driving …  I thought I’d look ridiculous”* ***(Archie)***

## Discussion

The findings of this study provide a rich insight into the lived experiences of individuals with CLE and their photoprotective behaviours. Taking care in the sun is not a simplistic process. Photoprotective behaviours are complex, multi-layered and personal. Moreover, it is a process that is subject to a wide variety of mediating factors that can change with time and circumstances. The additional efforts of taking care in the sun might appear effortless to others, but for individuals living with CLE can be challenging. Beyond the *biomedical* model of illness, the *social* impact that CLE has on individuals has a dominant influence on their intent and willingness to adhere to photoprotective measures. Often there can be a greater focus on the *biomedical* model in healthcare, which can risk objectifying individuals with CLE and their photoprotective behaviours.

### Photoprotection: *More extraordinary than mundane … *

Adhering to photoprotective measures requires sustained changes to individual’s behaviours. Providing photoprotective advice is merely a starting point. Considering how to translate such advice into behavioural change is of at least equal importance. Behavioural change is a complex process, as evidenced by participants in this study. A number of behavioural frameworks have been espoused to make visible the steps necessary to guide behavioural change. One such model is the ‘behavioural change wheel’.^20^ In this model, the essential conditions for behavioural change are characterised as the *capability*, *motivation* and *opportunities* to support change and were consistent with our research.

#### Having the capability for change

In terms of the *capability* to take care in the sun, acquiring the knowledge of photoprotective measures is an important foundation. But having the capability to enact such measures is important, for example having the financial capability to buy photoprotective clothing. Inherent physical properties of sunscreen have previously been identified as a barrier to its use in individuals with CLE.^14^ This was also identified in this study, particularly in reference to the limited range of sunscreens available on prescription within the NHS. Thus, having the financial capability to purchase sunscreen that feels more pleasant on one’s skin is essential to facilitate the behavioural change of using sunscreen regularly.

#### Having the motivation for change

Evident from the experiences of the individuals in this study, *motivation* to adhere to photoprotective measures is important. Sustaining photoprotective behaviours requires effort and drive; however, the clinical burden of CLE can act as a barrier to adhering to such measures. Aside from practical barriers such as applying sunscreen to areas of inflamed skin, the associated psychological sequalae and systemic symptoms in those with co-existing SLE, such as pain and fatigue, can demotivate patients as indicated by the results of this study.

Furthermore, in the event of unpredicted social situations, individuals with CLE have to balance the extra effort of taking care in the sun with the real risks of sun exposure. Such a challenge mediates their motivational drive, and individuals often default to avoid the circumstances if possible or to ‘take the risk’ of sun exposure with suboptimal photoprotection. Previous experiences of the impact of sun exposure on their condition can also mediate their motivation. Whilst previous CLE flare-ups triggered by sun exposure can enhance their motivation, experiencing symptoms despite their best photoprotective efforts can also be demotivating.

#### Having the opportunities for change

Whilst having the *capability* and *motivation* to adhere to photoprotective advice, practical *opportunities* were also of importance to individuals, for example receiving sunscreen on prescription or attending outdoor social gatherings with shaded areas. Furthermore, the busy nature of life can challenge individual’s compliance with photoprotection. Individuals find themselves having to balance the need to practice photoprotection with other demands such as looking after young children or maintaining their garden. Unfortunately in situations like these, complying with photoprotective measures is not always prioritised.

### The social dimension and stigma of photoprotection

Without question, the social dimension of photoprotective behaviours plays a critical role in such individual’s lives. The disruption that CLE had on individual’s social worlds was a prominent experience. Individuals perceived that ‘enjoying the sun’ was a *social norm* and something that they had to contend with on a daily basis. Social norms can be defined as *‘rules and standards that are understood by members of a group, and that guide or constrain social behaviours’.*^21^ For individuals with CLE, prior to their development of CLE and photosensitivity, ‘enjoying’ the sun was a ‘norm’ for them. A diagnosis of CLE marked an important transition that required adaptation to a ‘new norm’ of striving to change their photoprotective behaviours. This sense of loss could be a challenge for such individuals, similar in ways to the five stages of grief as outlined by Kübler-Ross,^22^ and some would often retain a sense of always wanting to enjoy the sun again.

On face value, photoprotective measures such as applying sunscreen, wearing long sleeve clothing or a wide brimmed hat appear to be relatively simple. Despite this, as evidenced in this study, enacting such measures can be challenging and reinforce individuals feeling of being *different.* Patients with CLE may already struggle with feeling *different* from other members of society due to the presence of their condition on exposed sites. Certain photoprotective measures may exacerbate this sense of feeling *different* such as wearing long sleeve clothing outdoors when it’s sunny.

In the presence of others, who were unaware of their condition, individuals living with CLE would make efforts to appear *normal* and conceal their CLE. This included concealing photoprotective practices not in keeping with social norms. In addition, in spontaneous social circumstances involving sun exposure, individuals would often recall sadness that they couldn’t readily adapt, resulting in social isolation. In contrast, when in the presence of others who were aware of their condition, individuals were often extended gestures of support, which promoted a motivation to adhere to photoprotective advice, for example a friend providing shade at an outside gathering.

The Canadian sociologist Erving Goffman described stigma as *“the phenomenon whereby an individual with an attribute which is deeply discredited by his/her society is rejected as a result of the attribute. Stigma is a process by which the reaction of others spoils normal identity”.*^23^ The experience of both having CLE and implementing photoprotective practices could be considered discrediting experiences. This sense of *otherness* was largely unwelcomed by individuals with CLE who often took steps to conceal their condition or photoprotective efforts. Society exerts expectations of what is considered to be ‘*normal’* and ‘*abnormal’.* Such experiences of not being ‘*normal’* are supported by the notion that stigma is often defined and reinforced through social interactions.^23^ Despite prior documentation that individuals with CLE may experience stigma for exercising extra photoprotective efforts,^24^ this study is the first to establish that this stigma can inhibit such individuals practice of photoprotection. As CLE is relatively rare, perhaps increasing awareness of the condition and its photoprotective requirements at a societal level is not the most effective way to manage the stigma experienced by individuals with CLE. Instead offering individuals support to help them manage this felt stigma may be more effective. Previous research has found success in increasing enabling skills of individuals with SLE through a self-help course,^25,26^ and so perhaps a similar course could be created or recommended for individuals with CLE who struggle to overcome the adversities associated with their condition.

### Strengths and limitations

Despite the many strengths of this study, it has to be considered within its limitations. Given the conceptual orientation of this study, generalisability was not an intended aim. This study was exploratory, shedding light on the fine-grained experiences of individuals with CLE and their photoprotective behaviours, and findings may be more transferable than generalizable to the wider CLE community. In this study, the majority of individuals were female in keeping with the fact that women are more likely than men to develop CLE. Gender influences on photoprotective behaviours were not specifically explored but would be worthy of future research. Lastly, this study did not set out to measure the impact of interventions on photoprotective behavioural change. Despite these limitations, this research provides a novel insight into the experiences of individuals living with CLE and their photoprotective behaviours that can serve to guide future behavioural interventional/quantitative studies and importantly help inform consultations with CLE patients in the clinical setting.^27^

## Conclusions

Photoprotection is central to the effective management of CLE. This study provides a deep, nuanced insight into the lives of individuals with CLE and their experiences of photoprotection. Such privileged insights provide a foundation of how best to help individuals living with CLE optimise their photoprotective behaviours. Sustained photoprotective behaviours are complex and multifaceted. On face value, and even to some healthcare professionals, photoprotective measures can be perceived as a simplistic process. This is not the case, as illustrated by the experiences of the individuals in this study. The disruption and extra burden that CLE places on such individual’s lives is important to be cognisant of in promoting photoprotective practices. Often healthcare professionals have a greater focus on disease (healthcare professionals definition of health problems) rather than illness experiences (individual subjective experiences of the disruption that health problems can have on them as a person).^28^ Recognising the social and psychological implications of photoprotection can help to guide a more person-centred approach for healthcare professionals in optimising photoprotection behaviours. Knowing such lived experiences reinforces one’s understanding that taking care in the sun with CLE is more than *skin deep.*

## Supplemental Material

sj-pdf-1-lup-10.1177_0961203320958067 - Supplemental material for ‘More extraordinary than mundane … ’ A phenomenological analysis of the experiences of individuals living with CLE and their taking care in the sun Click here for additional data file. Supplemental material, sj-pdf-1-lup-10.1177_0961203320958067 for ‘More extraordinary than mundane … ’ A phenomenological analysis of the experiences of individuals living with CLE and their taking care in the sun by Bláithín McGarry, Donal O'Kane, Collette McCourt and Gerard J Gormley in Lupus

## References

[1] Tan EM Cohen AS Fries JF, et al The 1982 revised criteria for the classification of systemic lupus erythematosus. Arthritis Rheum 1982; 25: 1271–1277. 7138600 10.1002/art.1780251101

[2] Durosaro O Davis MDP Reed KB Rohlinger AL. Incidence of cutaneous lupus erythematosus, 1965-2005: a population-based study. Arch Dermatol 2009; 145: 249–253. 19289752 10.1001/archdermatol.2009.21 PMC3953616

[3] Klein R Moghadam-Kia S Taylor L, et al Quality of life in cutaneous lupus erythematosus. J Am Acad Dermatol 2011; 64: 849–858. 21397983 10.1016/j.jaad.2010.02.008 PMC3079065

[4] Vasquez R Wang D Tran QP, et al A multicentre, cross-sectional study on quality of life in patients with cutaneous lupus erythematosus. Br J Dermatol 2013; 168: 145–153. 22708924 10.1111/j.1365-2133.2012.11106.x PMC3467361

[5] Kim A Chong BF. Photosensitivity in cutaneous lupus erythematosus. Photodermatol Photoimmunol Photomed 2013; 29: 4–11. 23281691 10.1111/phpp.12018 PMC3539182

[6] Rutter KJ Ashraf I Cordingley L Rhodes LE. Quality of life and psychological impact in the photodermatoses: a systematic review. Br J Dermatol 5: 1092–1102. 10.1111/bjd.18326 31278744

[7] Yang SY Bernstein I Lin DQ Chong BF. Photoprotective habits of patients with cutaneous lupus erythematosus. J Am Acad Dermatol 2013; 68: 944–951. 23360867 10.1016/j.jaad.2012.11.016 PMC3657339

[8] Schneider J. The teaspoon rule of applying sunscreen. JAMA Dermatol 2002; 138: 838–839. 10.1001/archderm.138.6.838-b 12056975

[9] Kuhn A Gensch K Haust M, et al Photoprotective effects of a broad-spectrum sunscreen in ultraviolet-induced cutaneous lupus erythematosus; randomized, vehicle-controlled, double-blind study. J Am Acad Dermatol 2011; 64: 37–48. 21167404 10.1016/j.jaad.2009.12.053

[10] Zahn S Graef M Patsinakidis N, et al Ultraviolet light protection by a sunscreen prevents interferon-driven skin inflammation in cutaneous lupus erythematosus. Exp Dermatol 2014; 23: 516–518. 24758584 10.1111/exd.12428

[11] Stege H Budde M-A Grether-Beck S Krutmann J. Evaluation of the capacity of sunscreens to photoprotect lupus erythematosus patients by employing the photoprovocation test. Photodermatol Photoimmunol Photomed 2000; 16: 256–259. 11132128 10.1034/j.1600-0781.2000.160604.x

[12] Patsinakidis N Wenzel J Landmann A, et al Suppression of UV-induced damage by a liposomal sunscreen: a prospective, open-label study in patients with cutaneous lupus erythematosus and healthy controls. Exp Dermatol 2012; 21: 958–961. 23171460 10.1111/exd.12035

[13] Cusack C Danby C Fallon JC, et al Photoprotective behaviour and sunscreen use: impact on vitamin D levels in cutaneous lupus erythematosus. Photodermatol Photoimmunol Photomed 2008; 24: 260–267. 18811868 10.1111/j.1600-0781.2008.00373.x

[14] Gutmark EL Lin DQ Bernstein I Wang SQ Chong BF. Sunscreen use in patients with cutaneous lupus erythematosus. Br J Dermatol 2015; 173: 831–834. 25703638 10.1111/bjd.13736 PMC4545747

[15] Tong A Sainsbury P Craig J. Consolidated criteria for reporting qualitative research (COREQ): a 32-item checklist for interviews and focus groups. Int J Qual Health Care 2007; 19: 349–357. 17872937 10.1093/intqhc/mzm042

[16] Smith JA Flowers P Larkin M. Interpretative phenomenological analysis: theory, method and research. Thousand Oaks: SAGE Publications Ltd, 2009.

[17] Smith JA Osborn M. Interpretative phenomenological analysis In: Smith JA (ed.) Qualitative psychology: a practical guide to research methods. 2nd ed Thousand Oaks: SAGE Publications Ltd, 2007, pp.25–52.

[18] Booton CM. Using rich pictures to verify, contradict, or enhance verbal data. Qual Rep 2018; 23: 2835–2849.

[19] Brooks J McCluskey S Turley E King N. The utility of template analysis in qualitative psychology research. Qual Res Psychol 2015; 12: 202–222. 27499705 10.1080/14780887.2014.955224 PMC4960514

[20] Michie S Van Stralen MM West R. The behaviour change wheel: a new method for characterising and designing behaviour change interventions. Implement Sci 2011; 6: 42. 21513547 10.1186/1748-5908-6-42 PMC3096582

[21] Cialdini R Trost M. Social influence: Social norms, conformity and compliance In: Gilbert D Fiske S Lindzey G (eds) The handbook of social psychology. 4th ed. New York: McGraw-Hill, 1998, p.152.

[22] Kübler-Ross E Kessler D. On grief and grieving. New York: Scribner, 2005, pp.7–28.

[23] Goffman E. Stigma: notes on the management of spoiled identity. Upper Saddle River: Prentice-Hall, 1963.

[24] Hale ED Treharne GJ Norton Y, et al ‘ Concealing the evidence’: the importance of appearance concerns for patients with systemic lupus erythematosus. Lupus 2006; 15: 532–540. 16942007 10.1191/0961203306lu2310xx

[25] Sohng KY. Effects of a self-management course for patients with systemic lupus erythematosus. J Adv Nurs 2003; 42: 479–486. 12752868 10.1046/j.1365-2648.2003.02647.x

[26] Braden CJ McGlone K Pennington F. Specific psychosocial and behavioral outcomes from the systemic lupus erythematosus self-help course. Health Educ Q 1993; 20: 29–41. 8444623 10.1177/109019819302000105

[27] Servive O Hallsworth M Halpern D, et al EAST: four simple ways to apply behavioural insights. The Behavioural Insights Team in partnership with the UK Cabinet Office and Nesta. Report number: 1, 2014.

[28] Carel HH. Illness, phenomenology, and philosophical method. Theor Med Bioeth 2013; 34: 345–357. 23836135 10.1007/s11017-013-9265-1

